# Mortality after benign thyroid surgery in patients aged 80 years or older

**DOI:** 10.1007/s00423-022-02463-2

**Published:** 2022-03-14

**Authors:** Salem A. Farhad, Bergenfelz Anders, Nordenström Erik, Nilsson Martin, Almquist Martin

**Affiliations:** 1 Department of Clinical Sciences, Lund University, Lund, Sweden; 2 Skåne University Hospital, 221 85 Lund, Sweden

**Keywords:** Mortality, Benign thyroid surgery, Elderly

## Abstract

**Introduction:**

A recent report from the United Nations showed that aged people are increasing worldwide. Few data exist on overall survival for patients 80 years or older undergoing benign thyroid surgery. Short- and long-term survival and risk factors for death in patients undergoing thyroid surgery for benign disease were evaluated, using a nationwide, population-based quality register.

**Methods:**

Patients operated for benign thyroid disease, 2004 to 2017, were collected from the national quality register for thyroid surgery. Mortality data were retrieved from the Swedish National Board of Health and Welfare. Mortality at 30 days, 90 days, and 1 year after surgery, for patients 80 years or older, was calculated. Overall survival was calculated using the Kaplan–Meier estimate. Risk factors for mortality were assessed with Cox’s multiple regression analysis. The standardized mortality ratio was calculated.

**Results:**

There were 17,969 patients. Among them, 483 patients were 80 years or older, and of these, 397 (82.2%) were women. The mortality rate at 0–30 days, 31–90 days, and at 91–365 days after surgery was 0.4%, 0.2%, and 2.5%, respectively. The median (IQR) follow-up time was 4.5 (2.9–7.2) and the median (IQR) survival time was 8.0 (4.1–12.5) years. Apart from age, there was no other risk factor for death. The standardized mortality ratio (SMR) was 0.67 (0.49–0.91) for men and 0.76 (0.65–0.89) for women.

**Conclusion:**

Mortality after surgery for benign thyroid disease in patients 80 years or older was lower than the general population with no specific risk factors for death except for age.

## Introduction

With more people living long healthy lives, the number of patients undergoing surgery aged 80 years or older is increasing [1]. The United Nations expects the number of people aged 80 years or older to increase almost threefold from 2019 to 2050 worlwide [2]. In 2019, the largest number of people aged 80 years or older were found in Europe and Northern America. By 2050, East and South-East Asia are expected to have more than half of the world’s population aged 80 years and older [2]. While being older makes surgery more likely, it may also increase the potential risks for postoperative morbidity [3, 4] and mortality [5, 6]. In aged patients, surgery should maintain the life span and enhance the quality of life [7].

Operation for benign thyroid disease is common [8] in elderly patients. Studies performed on the quality of life after thyroidectomy for benign thyroid diseases, e.g., goiter with compression symptoms [9, 10] and inflammatory thyroid diseases [11, 12] show some benefits to the patients’ life quality. Some studies performed on the safety of thyroid surgery in elderly patients emphasize that high age is not a contraindication [13, 14]. In contrast, a large study performed by Echanique KA et al. [15] on 414,079 patients in which 33,646 patients were over the age of 75 years, showed that with the advanced age the thyroidectomy-related complications increased. Also, studies performed by Weiss et al. [16] on 150,012 patients and Sosa J.A. et al. [17] on 22,848 patients showed that older patients are at a higher risk for postoperative morbidity and in-hospital mortality than young patients. These and other studies [18–20] included patients with thyroid malignancy. Thus, data on mortality after thyroid surgery in older patients are conflicting. Therefore, we evaluated short- and long-term survival, risk factors for death, and standardized mortality ratio for patients aged 80 years or older undergoing thyroid surgery with benign histopathology.

## Material and methods

### Data collection

Data for thyroid operations performed between January 1, 2004, and December 31, 2017, were collected from the Scandinavian Quality Register for Thyroid, Parathyroid, and Adrenal Surgery (SQRTPA). This register has a coverage of over 90% in Sweden [21]. Data for death and cause of death from January 1, 2004, to June 30, 2019, were collected from the National Board of Health and Welfare. This register has coverage of almost 100% [22]. The two databases were then cross-matched. The standardized mortality rate for the general Swedish population aged 80 years or older was calculated using data from the Central Bureau of Statistics (SCB) and compared to the mortality rate in the present study. Patients below the age of 18 years, patients operated with an indication of malignancy, postoperative histopathological diagnosis of malignancy, thyroid malignancy as a cause of death, previous thyroidectomy, concomitant lymph node dissection, parathyroid surgery, operations other than thyroidectomy, and thyroid resection, and patients with data entry error were excluded from the study cohort.

### Statistical analysis

Patients aged 80 years or older were compared with the rest of the cohort regarding baseline characteristics and mortality rate for 30, 31–90, and 91–365 days after thyroid surgery. Continuous variables are reported as the median and interquartile range (IQR); categorical variables are reported as percentages. For comparison of continuous variables, the Wilcoxon rank-sum test was used, and for comparison of categorical variables, the chi-square test was used. A *p*-value of ≤ 0.05 was considered significant. Patients were followed from the date of surgery until death or end of follow-up on June 30, 2019. Loss-to-follow-up was not deemed to be a problem in this population and was not accounted for. Median follow-up was calculated using time-on-study, i.e., time from operation until death or end-of-follow-up. Mortality at 30, 31–90, and 91–365 days after surgery was calculated and compared to patients aged 18–79 years old. Overall survival was calculated using the Kaplan–Meier estimate. Risk factors for mortality were assessed with Cox’s multiple regression, adjusting for age, gender, indication for operation, substernal goiter, operation time, gland weight, and postoperative bleeding requiring re-operation that has shown to be associated with higher mortality [16]. Standardized mortality ratio (SMR) was calculated using the mortality rate from the cohort and the mortality rate in the general population during the same time span as the study population.

All analyses were performed using STATA/IC version 16.1, StataCorp LP, College Station, TX, USA.

## Results

The number of patients who had thyroid surgery for benign thyroid disease during the study period was 17,969. Among them, 483 (2.7%) patients were aged 80 years or older, 397 (82.2%) women. Patients aged 80 years or older were more often operated for compression symptoms and for excluding malignancy 302 (62.5%) and 139 (28.8%), respectively compared with patients aged 18–79 years, 8091 (46.3%) and 3730 (21.3%), *p* < 0.001. Unilateral procedures were more common in patients aged 80 years or older 312 (64.6%) compared with 9305 (53.2%) in patients aged 18–79 years, *p* < 0.001. Patients aged 80 years or older had more often a substernal goiter 154 (31.9%) compared with 1472 (8.4%) in patients aged 18–79 years, *p* < 0.001. Excised gland weight in patients aged 80 years or older was median (IQR) of 100 (30–171) g compared with 37.5 (20–81) g in patients aged 18–79 years. Histopathological diagnosis in patients aged 80 years or older was more often nodular goiter 375 (77.6%) compared with 9639 (55.1%) in patients aged 18–79 years, *p* < 0.001. Postoperative bleeding requiring re-operation was higher 15(3.1%) in patients aged 80 years and older compared with 267(1.5%) in patients aged 18–79 years, *p* = 0.02. Mortality rate at 30, 31–90, and 91–365 days after surgery for patients aged 80 years or older was 0.41%, 0.20%, and 2.5%, respectively and for patients aged 18–79 years 0.03%, 0.04%, and 0.2%, respectively, see Table 1.

**Table 1:** Characteristics of patients operated with thyroidectomy for benign thyroid disorder between 2004 and 2017. A comparison between patients 18–79 and 80 years or older

**Characteristics**	All cohort nr. 17,969 (100%)	Elderly (80 +) Nr. 483 (2.7%)	18—79 years nr. 17,486 (97.3%)	*p*-value
Gender				0.67†
Female	14,898 (82.9)	397 (82.2)	14,501 (82.9)	
Male	3071 (17.1)	86 (17.8)	2985 (17.1)	
Main indication for surgery				< 0.01†
Compressions symptoms	8393 (46.7)	302 (62.5)	8091 (46.3)	
Excluding malignancy	3869 (21.5)	139 (28.8)	3730 (21.3)	
Thyrotoxicosis	5530 (30.8)	37 (7.7)	5493 (31.4)	
Other indications	177 (1.0)	5 (1.0)	172 (1.0)	
Type of thyroid operation				< 0.01†
Bilateral procedure*	7969 (44.3)	156 (32.3)	7813 (44.7)	
Unilateral procedure**	9617 (53.5)	312 (64.6)	9305 (53.2)	
Isthmus resection	316 (1.8)	11 (2.3)	305 (1.7)	
Missing data	67 (0.4)	4 (0.8)	63 (0.4)	
Operation time in minutes median (IQR)				
Bilateral procedure	*120 (95–153)*	*109 (87–150)*	*120 (95–153)*	0.07††
Unilateral procedure	*80 (60–106)*	*80 (60–105)*	*80 (60–106)*	0.83††
Missing data	3207 (17.8)	78 (16.1)	3129(17.9)	
Substernal gland				< 0.01†
Yes	1626 (9.0)	154 (31.9)	1472 (8.4)	
No	14,081 (78.4)	267 (55.3)	13,814 (79.0)	
Missing	2262 (12.6)	62 (12.8)	2200 (12.6)	
Sternotomy				0.09†
Yes	66 (0.4)	4 (0.8)	62 (0.4)	
No	17,903 (99.6)	479 (99.2)	17,424 (99.6)	
Gland weight in grams median (IQR)	*38 (20.4–83.7)*	*100 (30–171)*	*37.5 (20–81)*	< 0.01††
Missing	4314 (24.1)	114 (23.4)	4,200 (24.1)	
Histopathological diagnosis				< 0.01†
Nodular goiter	10,014 (55.7)	375 (77.6)	9639 (55.1)	
Autoimmune inflammatory thyroid disease	4275 (23.8)	19 (4.0)	4256 (24.3)	
Benign tumor	2128 (11.9)	63 (13.0)	2065 (11.8)	
Other***	492 (2.7)	3 (0.6)	489 (2.8)	
Normal gland	98 (0.5)	2 (0.4)	96 (0.6)	
Missing data	962 (5.4)	21 (4.4)	941 (5.4)	
Postoperative complications:				
Bleeding requiring re-operation				
Yes	282 (1.6)	15 (3.1)	267 (1.5)	0.02†
No	17,602 (97.9)	467 (96.7)	17,135(98)	
Missing data	85 (0.5)	1 (0.2)	84 (0.5)	
Permanent vocal cord palsy				
Yes	120 (0.7)	7 (1.5)	113 (0.6)	0.10†
No	13,782 (76.7)	369 (76.4)	13,413 (76.7)	
Missing data	4067 (22.6)	107 (22.1)	3960 (22.7)	
Permanent hypoparathyroidism				
Yes	197 (1.1)	5 (1)	192 (1.1)	0.97†
No	13,737 (76.4)	371 (76.8)	13,366 (76.4)	
Missing data	4035 (22.5)	107 (22.1)	3928 (22.5)	
Surgical site infection				
Yes	174 (1)	8 (1.6)	166 (1)	0.11†
No	17,601 (98)	473 (98)	17,128 (98)	
Missing data	194 (1)	2(0.4)	192(1)	
Mortality				
Within 30 days after thyroid surgery	8 (0.04)	2 (0.41)	6 ( 0.03)	< 0.01†
Within 31–90 days after thyroid surgery	9 (0.05)	1 (0.20)	8 (0.04)	
Within 91–365.25 days after thyroid surgery	50 (0.27)	12 (2.5)	38 (0.2)	

Continuous variables are shown in italics

^††^The Wilcoxon rank-sum test

^†^The chi-square/*χ*^2^ test

^*^Total/near-total thyroidectomy and bilateral thyroid gland resections

^**^Hemithyroidectomy and unilateral thyroid gland resections

^***^Cysts and other thyroiditis than those caused by autoimmune disease

Apart from age, there was no other risk factor for death in the multivariable Cox regression analysis. Also, no correlation between gender, indication for surgery, substernal goiter, gland weight, operation time, and reoperation due to postoperative bleeding and survival was seen (see Table 2).

**Table 2:** Cox regression analysis of predictors for death after thyroidectomy for benign thyroid disease in patients aged 80 and older

Characteristics	Hazard ratio (95% CI)			
	Univariable	*p*-value	Multivariable	*p*-value
Age (years)				
80–85	1			
86–90	1.44 (1.01–2.04)	0.04	1.44 (1.00–2.07)	0.04
> 90	3.50 (2.13–5.74)	< 0.01	3.20 (1.92–5.32)	< 0.01
Gender				
Female	1			
Male	1.18 (0.83–1.67)	0.33	1.16 (0.81–1.67)	0.52
Indication for surgery				
Goiter with compression symptoms	1			
Excluding malignancy	0.91 (0.66–1.24)	0.56	1.03 (0.71–1.49)	0.88
Inflammatory thyroid disease	0.94 (0.54–1.63)	0.83	1.04 (0.58–1.85)	0.83
Other indication	0.82 (0.20–3.34)	0.79	0.91 (0.21–3.83)	0.93
Substernal goiter				
No	1			
Yes	1.26 (0.94–1.68)	0.16	1.23 (0.87–1.74)	0.33
Missing	0.60 (0.26–1.39)	0.23	0.63 (0.27–1.47)	0.29
Operation time				
Less than 2 h	1			
Over two hours	1.01 (0.72–1.42)	0.93	1.04 (0.72–1.49)	0.96
Missing	1.12 (0.78–1.60)	0.52	1.21 (0.83–1.75)	0.45
Gland weight				
Less than 200 g	1			
200–499 g	0.84 (0.55–1.27)	0.42	0.90 (0.58–1.38)	0.40
500 g and more	1.37 (0.43–4.34)	0.59	1.23 (0.37–4.09)	0.74
Missing	0.85 (0.60–1.20)	0.37	0.77 (0.54–1.10)	0.16
Postoperative bleeding requiring re-operation				
No	1			
Yes	1.04 (0.51–2.12)	0.89	0.93 (0.45–1.92)	0.84
Missing	5.43 (0.75–39.12)	0.09	6.41 (0.82–49.86)	0.07

The median interquartile range (IQR) follow-up time in patients aged 80 years or older was 4.5 (2.9–7.2) and the median (IQR) survival time for these patients was 8.0 (4.1–12.5) years. Patients aged 80 years or older had a lower death rate compared to people 80 years or older in the general Swedish population, SMR 0.67 (0.49–0.91) for men and 0.76 (0.65–0.89) for women.

## Discussion

In this nationwide, population-based study, short-term mortality in patients aged 80 years or older was low, and overall survival high during the median 4.5 years of follow-up after surgery for benign thyroid disease.

**Fig. 1:**
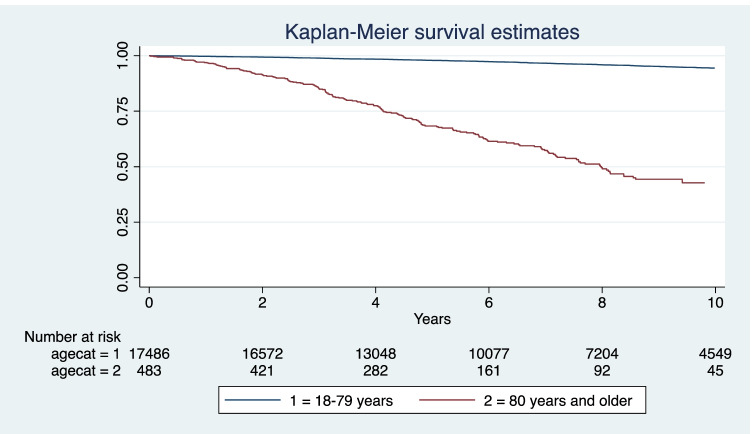
The Kaplan–Meier survival estimate comparing patients (18–79 years) with aged patients (80 years and older) after thyroid surgery

The short-term mortality was lower than those reported by Weiss et al. [23] and Sosa et al. [17]. These figures show that thyroid surgery for benign disease, also in patients aged 80 years or older, is indeed very safe (Fig. 1).

The median survival of 4.5 years after thyroid surgery for benign disease was almost similar to a report showing the expected survival of people of 85 years of age in Sweden [24]. This shows that thyroid surgery for benign disease, also in patients aged 80 years or older, is worthwhile if the surgery is expected to decrease symptoms and improve quality of life.

The low SMR in the study indicates that patients aged 80 years or older undergoing benign thyroid surgery are selected and that they are healthier than the rest of the population.

Echanique KA et al. [15] studied age-related complication in a very large number of patients who underwent thyroidectomy. In the study, 33,646 patients were 75 years and older. In-hospital mortality was higher in these patients than the younger patients. However, the study does not clarify mortality specifically in patients with benign thyroid disease which could differ from patients with malignant thyroid disease.

Compared with other types of surgeries, e.g., benign surgery [25, 26] and malignant surgery [27] in patients aged 80 years or older, thyroid surgery is considered safe [28]. However, the state of health and the preoperative comorbidities of these patients play a crucial role in the postoperative outcomes. Therefore, an individual risk–benefit analysis and careful preoperative preparation is key to successful postoperative outcome. [29].

Postoperative complications, e.g., bleeding has in a previous study by Weiss A. et al. [16] shown an association with higher mortality. However, in this study, we did not find an association.

With a noticeable increase in the aged population worldwide, there is a need to improve surgical care to maximize the positive outcomes and to minimize the burden of surgical comorbidities for the aged patient. The American College of Surgeons has recently introduced a geriatric surgery verification program suggesting standards for hospitals to provide better perioperative care for the aged and frail patients undergoing surgery [30]. One of the standards in the program is to advance the knowledge in geriatric surgery encouraging scholarly research in the improvement of the quality of geriatric surgery [31].

Limitations of the study include a lack of detailed data on comorbidities of the patients, as well as smoking, and other potential risk factors, which would have provided insights into patient selection. The strength of the study is that nationwide databases with the coverage of over 90% for thyroid surgery and almost 100 percent for population mortality are used.

## Conclusion

We conclude that age 80 years or older is not a contraindication in selected patients for thyroid surgery for benign disease with low short-term mortality and high overall survival.

## References

[1] Fowler AJ, Abbott TEF, Prowle J, Pearse RM (2019). Age of patients undergoing surgery. Br J Surg.

[2] United Nations, Department of Economic and Social Affairs, Population Division (2017) World Population Ageing 2017 - Highlights (ST/ESA/SER.A/397). 2017. Accessed 2017.

[3] Bergenfelz A, Jansson S, Kristoffersson A (2008). Complications to thyroid surgery: results as reported in a database from a multicenter audit comprising 3,660 patients. Langenbeck’s archives of surgery.

[4] Massarweh NN, Legner VJ, Symons RG, McCormick WC, Flum DR. Impact of advancing age on abdominal surgical outcomes. *Archives of surgery (Chicago, Ill : 1960).* 2009;144(12):1108–1114. 10.1001/archsurg.2009.204 20026827

[5] Katlic MR (2010). Consider surgery for elderly patients. CMAJ.

[6] Finlayson EV, Birkmeyer JD (2001). Operative mortality with elective surgery in older adults. Eff Clin Pract.

[7] Seybt MW, Khichi S, Terris DJ (2009). Geriatric thyroidectomy: safety of thyroid surgery in an aging population. Arch Otolaryngol Head Neck Surg.

[8] Mekel M, Stephen AE, Gaz RD, Perry ZH, Hodin RA, Parangi S (2009). Thyroid surgery in octogenarians is associated with higher complication rates. Surgery.

[9] Sorensen JR, Markoew S, Døssing H, Hegedüs L, Bonnema SJ, Godballe C (2018). Changes in swallowing symptoms and esophageal motility after thyroid surgery: a prospective cohort study. World J Surg.

[10] Sorensen JR, Watt T, Cramon P (2017). Quality of life after thyroidectomy in patients with nontoxic nodular goiter: a prospective cohort study. Head Neck.

[11] Kus LH, Hopman WM, Witterick IJ, Freeman JL (2017). Quality-of-life outcomes in Graves disease patients after total thyroidectomy. Ear Nose Throat J.

[12] Angell TE (2019). Thyroidectomy improves quality of life and fatigue in patients with Hashimoto’s disease and persistent symptoms compared to adequate thyroid hormone replacement. Clinical Thyroidology.

[13] Bliss R, Patel N, Guinea A, Reeve TS, Delbridge L (1999). Age is no contraindication to thyroid surgery. Age Ageing.

[14] Gervasi R, Orlando G, Lerose MA (2012). Thyroid surgery in geriatric patients: a literature review. BMC Surg.

[15] Echanique KA, Govindan A, Mohamed OM (2019). Age-related trends of patients undergoing thyroidectomy: analysis of US inpatient data from 2005 to 2013. Otolaryngol Head Neck Surg.

[16] Weiss A, Lee KC, Brumund KT, Chang DC, Bouvet M (2014). Risk factors for hematoma after thyroidectomy: results from the nationwide inpatient sample. Surgery.

[17] Sosa JA, Mehta PJ, Wang TS, Boudourakis L, Roman SA (2008). A population-based study of outcomes from thyroidectomy in aging Americans: at what cost?. J Am Coll Surg.

[18] Ng SH, Wong KP, Lang BH (2012). Thyroid surgery for elderly patients: are they at increased operative risks?. J Thyroid Res.

[19] Inversini D, Morlacchi A, Melita G (2017). Thyroidectomy in elderly patients aged >/=70 years. Gland Surg.

[20] Gomez-Ramirez J, Sitges-Serra A, Moreno-Llorente P (2015). Mortality after thyroid surgery, insignificant or still an issue?. Langenbeck’s archives of surgery.

[21] Annual Report (2017) Scandinavian quality register for thyroid, parathyroid and adrenal surgery. https://www.sqrtpa.se/arsrapporter

[22] Brooke HL, Talbäck M, Hörnblad J (2017). The Swedish cause of death register. Eur J Epidemiol.

[23] Weiss A, Parina RP, Tang JA, Brumund KT, Chang DC, Bouvet M (2015). Outcomes of thyroidectomy from a large California state database. Am J Surg.

[24] Statistics Sweden, Demographic report 2016:2, Life expectancy and mortality in different social groups. page 129. 2016

[25] Söderström L (2018) Differences in mortality among hipfracture patients in the Swedish Fracture Register [Master Thesis]. Sahlgrenska Academy, Gothenburg. https://www.registercentrum.blob.core.windows.net/sfr/r/Mortalitet-efter-ho-ftfraktur-Bke54pWNJN.pdf

[26] Jawad Z, Nemes S, Bülow E, Rogmark C, Cnudde P (2019). Multi-state analysis of hemi- and total hip arthroplasty for hip fractures in the Swedish population-Results from a Swedish national database study of 38,912 patients. Injury.

[27] Xu Y, Wang Y, Xi C, Ye N, Xu X (2019). Is it safe to perform gastrectomy in gastric cancer patients aged 80 or older?: A meta-analysis and systematic review. Medicine (Baltimore).

[28] Wong EH, Smith M, Fish B et al (2019) Thyroidectomy in octogenarians is not associated with poorer postoperative outcomes. Head Neck 41(8):2500–2506. 10.1002/hed.25712 10.1002/hed.25712 30828928

[29] Passler C, Avanessian R, Kaczirek K, Prager G, Scheuba C, Niederle B (2002). Thyroid surgery in the geriatric patient. Arch Surg (Chicago, Ill : 1960).

[30] Cooper L, Abbett SK, Feng A (2020). Launching a geriatric surgery center: recommendations from the Society for Perioperative Assessment and Quality Improvement. J Am Geriatr Soc.

[31] The American College of Surgeons (2019) Geriatric surgery verification program. https://www.facs.org/Quality-Programs/geriatric-surgery/standards

